# Human Cytokine Genetic Variants Associated With HBsAg Reverse Seroconversion in Rituximab-Treated Non-Hodgkin Lymphoma Patients: Erratum

**DOI:** 10.1097/01.md.0000484044.28366.8c

**Published:** 2016-05-20

In the article “Human Cytokine Genetic Variants Associated With HBsAg Reverse Seroconversion in Rituximab-Treated Non-Hodgkin Lymphoma Patients”,^1^ which appeared in Volume 95, Issue 11 of *Medicine*, an error was detected in Table 1. The article has since been corrected online.
